# The Impact of Neonatal Sepsis on Long-Term Neurodevelopment: A Systematic Review of Cognitive and Sensory Outcomes

**DOI:** 10.7759/cureus.90176

**Published:** 2025-08-15

**Authors:** Sara Mohamedosman Mohamed Ahmed Hamid, Kamal Shaban Ibrahim Shaban, Solafa Babeker Abdulhamid Abdulaziz, Sanaa Yosif Mohamed Ahmed Mohamed, Safwa Khatme Abdo Osman, Essra Tajelsir Abd Alnour Suliman

**Affiliations:** 1 Pediatrics, Dr. Sulaiman Al-Habib Medical Group, Riyadh, SAU; 2 Pediatrics, Southend University Hospital, Essex, GBR; 3 Pediatrics, Jazan General Hospital, Jazan, SAU; 4 Pediatrics, Maternity and Children Hospital, Al-Kharj, SAU; 5 Pediatrics, Mullingar Regional Hospital, Mullingar, IRL; 6 Pediatrics, Royal Surrey County Hospital, Guildford, GBR

**Keywords:** cognitive outcomes, motor impairments, neonatal sepsis, neurodevelopment, sensory deficits, systematic review

## Abstract

This systematic review examines the long-term cognitive and sensory outcomes associated with neonatal sepsis. A comprehensive literature search was conducted in PubMed, Web of Science, Scopus, and Embase from January 2020 to July 2025, following PRISMA (Preferred Reporting Items for Systematic Reviews and Meta-Analyses) guidelines. Studies were included if they reported neurodevelopmental outcomes among survivors of neonatal sepsis. A total of 14 eligible studies were identified and evaluated for risk of bias using the Newcastle-Ottawa Scale. Due to methodological heterogeneity, a narrative synthesis was performed. The included studies investigated a range of neurodevelopmental domains, with particular attention to cognitive, motor, and sensory outcomes. Findings varied across study populations and settings. Most studies reported a low risk of bias. This review highlights the need for standardized definitions of neonatal sepsis and outcome measures, as well as increased research focus on sensory outcomes and data from low-resource settings. Further prospective studies are recommended to enhance understanding of risk stratification and inform long-term follow-up strategies.

## Introduction and background

Neonatal sepsis is a critical global health concern, affecting an estimated three million newborns annually and contributing to approximately 225,000 neonatal deaths worldwide, with mortality rates ranging from 11% in high-income settings to over 40% in low-resource regions [1]. It disproportionately impacts preterm and low-birth-weight infants, accounting for a significant share of global neonatal morbidity and mortality. Characterized by systemic infection within the first 28 days of life, neonatal sepsis is classified into early-onset (≤72 hours) and late-onset (>72 hours) forms, each with distinct etiological and risk profiles [2]. Despite advances in neonatal care, survivors remain at risk for long-term neurodevelopmental impairments, here defined as deficits persisting beyond two years of corrected age, arising from the inflammatory cascade associated with sepsis, which can lead to white matter injury, neuronal damage, and disrupted brain maturation [3]. These pathological changes may manifest as cognitive deficits, motor impairments, and sensory processing disorders in early childhood and adolescence, underscoring the need for a deeper understanding of the condition's enduring consequences [4].

The mechanisms linking neonatal sepsis to adverse neurodevelopmental outcomes involve complex interactions between systemic inflammation, hypoxia-ischemia, and direct microbial toxicity [5]. Pro-inflammatory cytokines, microglial activation, and blood-brain barrier disruption can impair neurogenesis and synaptic connectivity, potentially resulting in conditions such as cerebral palsy, intellectual disability, and learning difficulties [6]. Additionally, sensory impairments, including hearing loss (often associated with ototoxic treatments or hypoxic injury) and visual deficits (due to retinopathy or cortical damage), further compound the developmental challenges faced by survivors [7]. However, the extent and severity of these outcomes vary widely across studies, influenced by sepsis severity, gestational age, and the quality of postnatal care, highlighting the need for a systematic synthesis of existing evidence.

Previous reviews have examined the association between neonatal sepsis and neurodevelopment, but most have either focused on composite neurodevelopmental impairment without separately addressing cognitive and sensory outcomes, restricted follow-up to infancy, or included heterogeneous populations combining term and preterm infants without stratification. This systematic review addresses these gaps by consolidating current knowledge on cognitive and sensory outcomes in neonatal sepsis survivors with follow-up into early childhood and beyond. By analyzing cohort studies, longitudinal follow-ups, and standardized neurodevelopmental assessments, it aims to clarify inconsistencies in the literature, identify key risk factors influencing trajectories, and provide evidence to guide clinical practice, early intervention strategies, and family counseling. Ultimately, this work seeks to bridge gaps in understanding the enduring impact of neonatal sepsis and inform targeted strategies to improve long-term outcomes for affected children.

## Review

Methodology

Search Strategy

This systematic review was conducted following the Preferred Reporting Items for Systematic Reviews and Meta-Analyses (PRISMA) guidelines [8]. A comprehensive literature search was performed across four major electronic databases: PubMed, Web of Science, Scopus, and Embase. The search strategy combined controlled vocabulary and free-text terms related to "neonatal sepsis," "long-term neurodevelopment," "cognitive outcomes," and "sensory outcomes." Boolean operators were used appropriately to refine the search. Only studies published within the last five years, from January 1, 2020, to July 15, 2025, were included to ensure that the findings reflect the most recent clinical definitions, diagnostic criteria, and advances in neonatal care. The full search strategy is provided in Table 4 in the Appendices section. Additional manual searching of the reference lists of included studies was conducted to identify any relevant articles that may have been missed during database searching.

Eligibility Criteria

Studies were eligible for inclusion if they met the following criteria: (i) original peer-reviewed research articles, (ii) involving neonates diagnosed with sepsis, (iii) reporting at least one long-term neurodevelopmental outcome, including cognitive or sensory outcomes, (iv) having a follow-up duration sufficient to assess neurodevelopment beyond infancy, and (v) published in English between 2020 and 2025. Exclusion criteria were reviews, editorials, conference abstracts, case reports, preprints, and studies that did not differentiate outcomes specific to neonatal sepsis or did not report cognitive or sensory assessments as separate results.

Study Selection

All records retrieved from database searches were imported into EndNote (Clarivate, Philadelphia, Pennsylvania) for de-duplication. Two reviewers independently screened titles and abstracts to identify potentially relevant studies, followed by a full-text review to confirm eligibility based on the inclusion and exclusion criteria. Discrepancies between reviewers were resolved through discussion and consensus, and when necessary, a third reviewer was consulted to facilitate a final decision.

Data Extraction

A standardized data extraction form was developed to systematically extract relevant information from the included studies. Extracted data included first author, publication year, country and setting, study design, sample size, population characteristics, definition and diagnostic criteria of neonatal sepsis, sepsis type, follow-up duration, neurodevelopmental outcomes assessed, assessment tools used, key findings, and reported effect estimates with statistical significance. Data extraction was conducted independently by two reviewers to ensure accuracy.

Risk of Bias Assessment

The risk of bias in the included studies was assessed using the Newcastle-Ottawa Scale (NOS) [9] for cohort and case-control studies, which evaluates methodological quality based on three domains: selection of participants, comparability of groups, and ascertainment of outcomes. Each study was independently rated by two reviewers, and any disagreements were resolved through consensus. Studies with higher NOS scores were considered to have a lower risk of bias.

Data Synthesis

Due to substantial heterogeneity in the included studies in terms of study designs, population characteristics, definitions of neonatal sepsis, outcome measures, assessment tools, and follow-up durations, it was determined that conducting a meta-analysis was not methodologically appropriate. Instead, a narrative synthesis was conducted to summarize the findings of the included studies systematically, focusing on cognitive and sensory outcomes associated with neonatal sepsis.

Results

Study Selection Process

**Figure 1:**
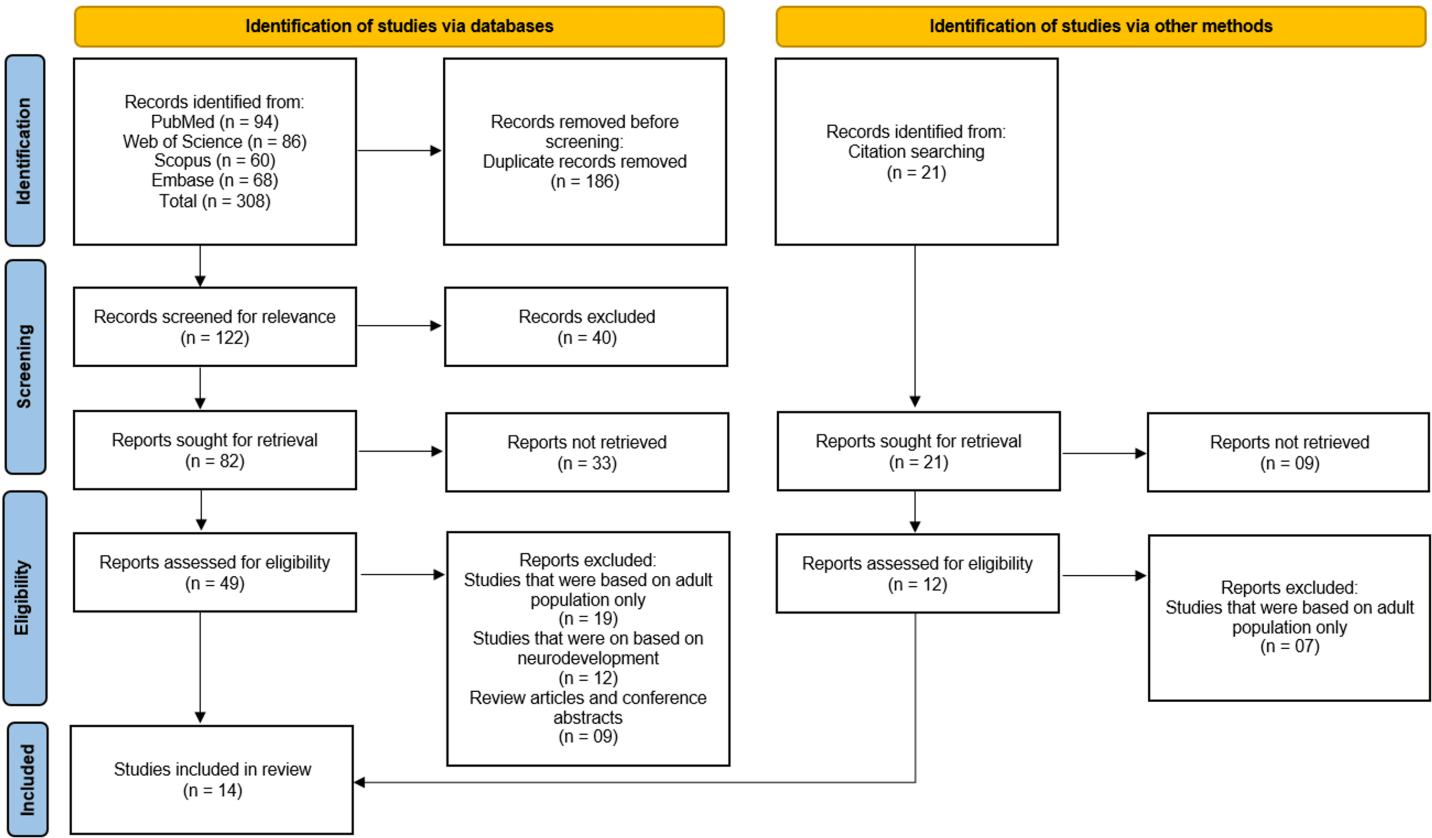
Illustration of the Studies Selection Process on the PRISMA Flowchart PRISMA: Preferred Reporting Items for Systematic Reviews and Meta-Analyses

The initial search across PubMed (n = 94), Web of Science (n = 86), Scopus (n = 60), and Embase (n = 68) yielded 308 records, supplemented by 21 additional studies identified through citation searching. After removing 186 duplicate records, 122 studies were screened for relevance, of which 40 were excluded. A total of 82 full-text reports were sought for retrieval, with 33 unavailable, leaving 49 articles assessed for eligibility. Of these, 19 were excluded for focusing solely on adult populations, 12 for lacking neurodevelopmental outcomes, and nine for being review articles or conference abstracts. Ultimately, 14 studies [10-23] met the inclusion criteria and were incorporated into the systematic review (Figure 1).

Study Characteristics

The systematic review included 14 studies [10-23] examining the long-term neurodevelopmental outcomes of neonatal sepsis, with diverse geographical settings and study designs. The studies were conducted in countries such as Pakistan, Canada, the Netherlands, South Africa, India, Mozambique, Kenya, Argentina, Kuwait, Germany, Denmark, Brazil, the USA, and South Korea. Study designs varied, including prospective and retrospective cohort studies, matched cohort studies, case-control studies, and cross-sectional analyses. Sample sizes ranged from 100 to 13,372 participants, with populations focusing on preterm infants, very low-birth-weight infants (VLBWIs), and term infants with a history of invasive bacterial infections. Neonatal sepsis definitions included pSBI (possible serious bacterial infection), culture-confirmed sepsis, and sepsis severity assessed using biomarkers or clinical scores, such as the nSOFA (neonatal Sequential Organ Failure Assessment). While culture-confirmed diagnosis was prioritized for its specificity and reduced misclassification risk, studies using standardized clinical criteria were also included to capture cases from settings where blood culture facilities are limited, thereby minimizing selection bias and ensuring representation of low-resource contexts. Follow-up durations spanned from infancy to early childhood (1.5-18 years), with neurodevelopmental outcomes assessed using tools such as the Bayley Scales of Infant Development, the Ten Questions Questionnaire (TQS), and standardized neurodevelopmental impairment (sNDI) criteria (Table 1).

**Table 1:** Characteristics of the Included Studies ANISA: Aetiology of Neonatal Infections in South Asia; pSBI: Possible Serious Bacterial Infection; WHO: World Health Organization; IMCI: Integrated Management of Childhood Illness; TQS: Ten Questions Screen; SDQ: Strengths and Difficulties Questionnaire; PEDS: DM-AL: Parent’s Evaluation of Developmental Stage Assessment Level; NICU: Neonatal Intensive Care Unit; CPBSI: Central Line-Associated Bloodstream Infection (CLABSI)/Catheter-Associated Bloodstream Infection; NR: Not Reported; sNDI: Severe Neurodevelopmental Impairment; NDI: Neurodevelopmental Impairment; GA: Gestational Age; nSOFA: Neonatal Sequential Organ Failure Assessment; CRP: C-Reactive Protein; PCT: Procalcitonin; IL-6: Interleukin-6; iGBS: Invasive Group B Streptococcus; EOS: Early-Onset Sepsis; LOS: Late-Onset Sepsis; ELBWI: Extremely Low-Birth-Weight Infant; MABC-2: Movement Assessment Battery for Children-2; LOM: Late-Onset Meningitis; VLBWI: Very Low-Birth-Weight Infant; PVL: Periventricular Leukomalacia; IUGR: Intrauterine Growth Restriction; NEC: Necrotizing Enterocolitis; MDI: Mental Development Index; PDI: Psychomotor Development Index; LOCNC: Late-Onset Culture-Negative Conditions; CSF: Cerebrospinal Fluid

First Author (Year)	Country/Setting	Study Design	Sample Size (n)	Population Characteristics	Neonatal Sepsis Definition	Sepsis Type	Follow-Up Duration	Neurodevelopmental Outcomes Assessed	Outcome Assessment Tools	Key Findings
Farheen et al., [10] (2024)	Pakistan/Karachi (ANISA study site)	Prospective follow-up cohort study	398 (241 cases, 157 controls)	Children with possible serious bacterial infection (pSBI) in early infancy assessed at 6–9 years of age compared to healthy controls	pSBI defined by the WHO IMCI algorithm	pSBI	6–9 years	Motor milestones, fine motor skills, receptive language, TQS milestones, hearing domain, self-help domain, SDQ-P overall score, anthropometry	Ten Questions (TQS) questionnaire, Strengths and Difficulties Questionnaire (SDQ), Parent’s Evaluation of Developmental Stage Assessment Level (PEDS: DM-AL)	pSBI was linked to motor delays, poorer fine motor skills, and abnormal TQS; receptive language was unaffected.
Zhou et al., [11] (2024)	Canada/26 tertiary-level NICUs in the Canadian Neonatal Network and Follow-Up Network	Retrospective cohort study	6,322	Infants <29 weeks gestational age admitted between 2010 and 2016	Late-onset CPBSI and late-onset meningitis	Late-onset sepsis (CPBSI), meningitis	NR	sNDI, neurodevelopmental impairment, cerebral palsy, cognitive, language, motor outcomes	Bayley-III scores	Increasing rates of adverse neurodevelopmental outcomes with no infection, CPBSI, and meningitis. IVH had an additive effect on outcomes.
Kurul et al., [12] (2023)	Netherlands	Retrospective cohort study	410	Very preterm neonates (GA <30 weeks), admitted 2016-2020	Sepsis episodes assessed using nSOFA score and inflammatory biomarkers (CRP, PCT, IL-6)	Sepsis episodes in preterm neonates; severity assessed by nSOFA ≥4	2 years corrected age	NDI, motor, and cognitive outcomes	Dutch Bayley Scales of Infant and Toddler Development (Bayley-III-NL)	Maximum CRP concentrations associated with lower motor and cognitive scores and increased risk of severe NDI; high nSOFA scores (≥4) associated with increased risk of mild NDI; no consistent association for IL-6 and PCT
Paul et al., [13] (2022)	South Africa, India, Mozambique, Kenya, Argentina	Multi-country matched cohort study	528 (138 iGBS survivors; 390 non-iGBS children)	Children aged 1.5–18 years with a history of invasive GBS disease (sepsis or meningitis) before 90 days of age; matched controls without GBS	History of invasive Group B Streptococcus disease (sepsis or meningitis) before 90 days	GBS-sepsis, GBS-meningitis	1.5–18 years	Cognitive, motor, hearing, vision, emotional-behavioral, and growth outcomes	Age-appropriate, culturally adapted assessments across multiple domains	38.1% of iGBS children had any NDI vs. 21.7% in controls; moderate/severe NDI: 15.0% and 5.6%; mild impairment more frequent in iGBS; emotional-behavioral risks similar between groups
Kartam et al., [14] (2022)	Kuwait/NICU of a maternity hospital	Retrospective cohort study	203	Preterm neonates, 24–32 weeks gestational age, admitted Jan–Dec 2017	Early-onset sepsis: ≤72 h postnatally; Late-onset sepsis (LOS): >72h postnatally	EOS and LOS	36 months corrected age	Motor, cognitive, and language composite scores	Bayley-III Scales of Infant Development	LOS linked to higher cerebellar hemorrhage risk and lower motor scores at 36 months; no adjusted cognitive/language associations.
Humberg et al., [15] (2022)	Germany	Multicenter observational cohort study	1343	ELBWI ≤28 6/7 weeks gestation, birth weight <1,000 g	Blood culture-proven late-onset sepsis (after day 7 of life)	LOS; recurrent sepsis (≥2 episodes)	5–6 years	Motor development, Intelligence Quotient, Behavioral difficulties	MABC-2 testing for motor development; standardized neurodevelopmental, behavioral, and motor tests by blinded investigators	Recurrent LOS linked to worse motor outcomes (1.7× higher odds); no effect on IQ or behavior.
Horváth-Puhó et al., [16] (2021)	Denmark and the Netherlands	Retrospective cohort study using national medical and administrative databases	2258 children with iGBS disease (1561 Denmark; 697 Netherlands) + 22,462 matched unexposed controls	Neonates ≤89 days with invasive GBS disease; matched unexposed controls by sex, birth year/month, gestational age	Culture-confirmed invasive GBS disease diagnosed by 89 days of age	Sepsis, meningitis, pneumonia (pneumonia only in Denmark)	Median 14 years (IQR 7–18) Denmark; median 9 years (IQR 6–11) Netherlands	NDIs; mortality	Denmark: discharge diagnoses in the National Patient Registry; Netherlands: special educational support records	iGBS meningitis linked to higher 5-year mortality; any iGBS raised the 10-year NDI risk. Exposed children ≤5 years had more clinic visits and admissions. Household income unaffected.
Horváth-Puhó et al., [17] (2022)	Denmark and the Netherlands (Nationwide cohorts)	Cohort study using national medical databases	487 preterm and 1642 term children with iGBS; 21,172 matched comparators	Preterm and term infants with a history of iGBS	iGBS identified via medical records	iGBS (bacterial sepsis)	Up to early childhood (mortality assessed by 3 months)	NDI	NR	Preterm iGBS infants showed high mortality (671/1000 person-years, Netherlands) and NDI risk (~9%, Denmark and Netherlands); prematurity plus iGBS greatly increased both risks.
Golin et al., [18] (2022)	Brazil	Prospective cross-sectional	100 (50 sepsis, 50 non-sepsis)	Preterm newborns (PTNBs), randomly selected	NR	NR	At the neonatal period	Neurological abnormalities (tone, tone patterns, reflexes, movements, abnormal signs, behavior)	Hammersmith Neonatal Neurological Examination (HNNE)	PTNBs with sepsis had significantly lower HNNE scores, indicating neurological dysfunction post-sepsis
Brumbaugh et al., [19] (2022)	USA (25 NICHD Neonatal Research Network centers)	Multicenter prospective cohort (secondary analysis)	13,372	Infants born at 22-26 weeks’ gestation (extremely preterm) between 2003 and 2017; median GA 25.4 weeks; 51% male	Culture-confirmed late-onset meningitis (LOM) and LOS	LOM and LOS	Neurodevelopmental follow-up at 18-26 months corrected age	Death or NDI	NR	LOM incidence decreased from 2% (2003) to 0.4% (2017). Both LOM (aOR 1.53) and LOS without LOM (aOR 1.41) were associated with increased risk of death or NDI.
Shim et al., [20] (2021)	South Korea/Korean Neonatal Network	Population-based cohort study	2,098	VLBWIs born at 23–32 weeks gestation between Jan 2014 and Dec 2017	Blood culture positive sepsis during hospitalization	LOS	18–24 months corrected age	Cognitive delay, Motor delay	Bayley Scales of Infant Development	LOS tied to cognitive delay only. IVH, PVL, IUGR, ventilation linked to both. Male sex, NEC ≥2 → motor delay; paternal education ↓ cognitive delay risk.
Ortgies et al., [21] (2021)	Germany	Cohort study	166	VLBWI (<1500 g), gestational age <35 weeks, born between 2008 and 2011	EOS defined as culture-proven or clinical EOS based on blood culture, CrP levels, and clinical symptoms/treatment	EOS	2 years corrected age	NDI, including MDI, PDI, and cerebral palsy	Bayley Scales of Infant Development-II	EOS impaired neurodevelopment at 2 years with higher NDI (24% vs. 8%); lower PDI (p=0.03); no MDI difference (p=0.77); chorioamnionitis and poor CRIB scores were risk factors.
Mukhopadhyay et al., [22] (2021)	USA/24 neonatal centers	Retrospective cohort study	3940	Infants born 2006–2014, gestational age 22–26 weeks, birth weight 401–1000 g, survived >7 days; excluded infants with early-onset sepsis, NEC, intestinal perforation, or both LOS and LOCNC	LOS: antibiotic administration ≥5 days with positive blood/CSF culture; LOCNC: antibiotic administration ≥5 days without positive culture	LOS, Late-onset culture-negative conditions (LOCNC)	18–26 months corrected age follow-up	NDI, death	NR	LOS raised the death risk but not the NDI versus LOCNC. Among survivors, NDI risk was similar, though LOCNC infants had higher NDI risk than unaffected ones. Further study of LOCNC causes and antibiotic impact is needed.
Nakwa et al., [23] (2020)	South Africa (3 secondary–tertiary hospitals in Johannesburg)	Case–control study	122 invasive GBS cases (78 sepsis, 44 meningitis) + 449 controls	Infants <90 days of age with invasive GBS disease; controls matched on maternal age, maternal HIV status, gestational age, timing of enrollment	Isolation of GBS from blood (sepsis) or CSF (meningitis) in infants <90 days	Sepsis and Meningitis (reported separately)	1 year	Neurologic impairment (overall developmental outcomes)	Denver II Developmental Screening Tool	GBS-sepsis survivors had 3.5-fold greater odds of neurologic impairment at 1 year compared to controls; meningitis cases also showed higher impairment rates, though not statistically significant.

Cognitive Outcomes

Neonatal sepsis was consistently associated with adverse cognitive outcomes across multiple studies. Farheen et al. [10] reported delayed TQS milestones in children with pSBI, though receptive language skills remained unaffected. Zhou et al. [11] found that very preterm infants with sepsis, particularly those with meningitis, had higher rates of sNDI, with cognitive scores <85 on the Bayley-III. Similarly, Kurul et al. [12] observed lower cognitive scores in preterm neonates with late-onset sepsis (LOS), particularly in cases with high nSOFA scores (≥4). Paul et al. [13] noted that iGBS (invasive group B *Streptococcus*) survivors had a higher risk of moderate/severe NDI (15.0% for meningitis, 5.6% for sepsis) compared to controls. Horváth-Puhó et al. [16] reported elevated risks of cognitive delays in Danish and Dutch cohorts, with a relative risk (RR) of 1.77 and 2.28, respectively, for NDIs at 10 years. Preterm iGBS infants faced even greater risks, with mortality and NDI rates as high as 671/1000 person-years in the Netherlands [17].

Motor Outcomes

Motor impairments were a prominent finding in neonates with sepsis. Farheen et al. [10] identified significant delays in motor milestones and fine motor skills in pSBI cases. Zhou et al. [11] linked sepsis and meningitis to lower Bayley-III motor scores and cerebral palsy. Kartam et al. [14] reported that LOS was associated with cerebellar hemorrhage and reduced motor scores at 36 months. Humberg et al. [15] found that recurrent LOS in extremely low birth weight infants (ELBWIs) increased the odds of motor deficits by 1.7-fold. Ortgies et al. [21] observed that early-onset sepsis (EOS) was associated with lower PDI scores and a 3.3-fold increased risk of NDI. Golin et al. [18] documented tone abnormalities and reflex impairments in preterm newborns post-sepsis, with 86% exhibiting neurological dysfunction.

Sensory Outcomes

Sensory outcomes, though less frequently reported, showed mixed results. Farheen et al. [10] found no significant differences in hearing domain scores using TQS, while Zhou et al. [11] noted language delays as a proxy for sensory deficits. Paul et al. [13] included hearing and vision assessments within NDI but did not provide separate effect estimates. Horváth-Puhó et al. [16] reported sensory impairments as part of NDIs but did not isolate specific sensory domains. Although evidence is limited, these findings suggest that sensory deficits, when present, may be under-recognized and potentially overlooked in follow-up care. This highlights the importance of incorporating routine hearing and vision screening into long-term follow-up protocols for neonatal sepsis survivors, as early identification and intervention could mitigate compounding effects on cognitive, language, and motor development.

Effect Estimates and Risk Factors

Adjusted analyses highlighted sepsis severity and comorbidities as key risk factors. Kurul et al. [12] demonstrated that higher nSOFA scores and CRP levels were associated with severe NDI (OR 1.01, 95% CI 1.00-1.01). Brumbaugh et al. [19] found that late-onset meningitis (LOM) increased the odds of death or NDI (aOR 1.53, 95% CI 1.04-2.25). Shim et al. [20] identified intraventricular hemorrhage (IVH), periventricular leukomalacia (PVL), and male sex as contributors to cognitive and motor delays. Ortgies et al. [21] linked chorioamnionitis and poor CRIB scores to worse neurodevelopmental outcomes in EOS cases.

Summary of Findings

The reviewed studies collectively underscore the detrimental impact of neonatal sepsis on long-term neurodevelopment, with cognitive and motor impairments being the most consistently reported outcomes (Table 2). Sensory deficits were less frequently examined but may contribute to broader NDIs. Preterm infants and those with severe sepsis or comorbid conditions (e.g., meningitis, IVH) faced the highest risks. These findings emphasize the need for early intervention and long-term follow-up for sepsis survivors to mitigate adverse outcomes.

**Table 2:** Neurodevelopmental and Sensory Outcomes Associated With Neonatal Sepsis TQS: Ten Questions Screen; SDQ: Strengths and Difficulties Questionnaire; PEDS: DM-AL: Parent’s Evaluation of Developmental Stage Assessment Level; sNDI: Severe Neurodevelopmental Impairment; CPBSI: Central Line-Associated Bloodstream Infection (CLABSI)/Catheter-Associated Bloodstream Infection; NR: Not Reported; NDI: Neurodevelopmental Impairment; Bayley-III: Bayley Scales of Infant and Toddler Development, Third Edition; Bayley-III-NL: Dutch version of Bayley-III; iGBS: Invasive Group B Streptococcus; CA: Corrected Age; MABC-2: Movement Assessment Battery for Children-2; RR: Risk Ratio; aHR: Adjusted Hazard Ratio; OR: Odds Ratio; HNNE: Hammersmith Neonatal Neurological Examination; LOM: Late-Onset Meningitis; LOS: Late-Onset Sepsis; IVH: Intraventricular Hemorrhage; PVL: Periventricular Leukomalacia; IUGR: Intrauterine Growth Restriction; NEC: Necrotizing Enterocolitis; EOS: Early-Onset Sepsis; MDI: Mental Development Index; PDI: Psychomotor Development Index; CRIB: Clinical Risk Index for Babies; LOCNC: Late-Onset Culture-Negative Conditions; aOR: Adjusted Odds Ratio

First Author (Year)	Cognitive Outcomes Reported	Motor Outcomes Reported	Sensory Outcomes Reported	Effect Estimates (95% CI)	Adjusted Covariates	Statistical Significance (p-value)
Farheen et al., [10] (2024)	Delayed TQS milestones; Receptive language skills (no significant difference); SDQ overall score	Greater delays in motor milestones; Lower fine motor skills	TQS hearing domain (no significant difference)	TQS milestones: β = -0.6, 95% CI -1.2 to -0.04; TQS hearing: β = -0.3, 95% CI -1.2 to 0.7; PEDS fine motor: β = -1.3, 95% CI -4.4 to 1.7; Receptive language: β = -1.1, 95% CI -3.7 to 1.4; Self-help domain: β = 0.6, 95% CI -1.2 to 2.4; SDQ overall: β = 0.02, 95% CI -0.3 to 0.3	Anthropometry (weight, height), sociodemographic variables	TQS abnormal scores p=0.001; Motor milestone delays p=0.02; Fine motor skills p=0.02
Zhou et al., [11] (2024)	Bayley-III cognitive scores <85; sNDI	Bayley-III motor scores <85; Cerebral palsy	Language scores <85 (as sensory proxy reported)	sNDI rates: No infection 15.0%, CPBSI 22.9%, Meningitis 32.0% (adjusted)	Infant characteristics	p < 0.05
Kurul et al., [12] (2023)	Lower cognitive scores on Bayley-III-NL	Lower motor scores on Bayley-III-NL	NR	Cognitive: −0.03 points (95% CI −0.06; −0.004) Motor: −0.03 points (95% CI −0.07; −0.00) Severe NDI: OR 1.01 (95% CI 1.00; 1.01) Mild NDI (nSOFA ≥4): OR 2.01 (95% CI 1.34; 3.03)	Gestational age, sex, birthweight-for-gestational-age SD score	Cognitive: p<0.05 Motor: p=0.05 severe NDI: p<0.05; mild NDI: p<0.05
Paul et al., [13] (2022)	NDI, including cognitive domain: Moderate/severe NDI risk in iGBS survivors was 15.0% (95% CI: 3.4–30.8%) for GBS-meningitis, 5.6% (95% CI: 1.5–13.7%) for GBS-sepsis survivors; mild impairment 27.6% (95% CI: 20.3–35.5%)	Motor outcomes were assessed within NDI	Hearing and vision assessed within NDI; no separate effect estimates reported for sensory outcomes	Adjusted risk ratio (aRR) for moderate/severe NDI: 1.27 (95% CI: 0.65–2.45); emotional-behavioral problems aRR=0.98 (95% CI: 0.55–1.77)	Adjusted for age and sex (matched cohort study design)	CI for aRR crosses 1, indicating not statistically significant
Kartam et al., [14] (2022)	Lower cognitive composite scores at 36 months CA (association not significant after adjustment)	Lower motor composite scores at 36 months CA (association remained significant after adjustment)	NR	Motor scores: adjusted β = –9.5 (95% CI: –16.4 to –2.7)	Gestational age, birth weight, cerebellar hemorrhage, white matter injury	Motor: p = 0.007; Cognitive: not significant after adjustment
Humberg et al., [15] (2022)	No significant impact on intelligence quotient reported	Adverse motor development (critical MABC-2 testing)	NR	OR 3.3 (1.5–7.3) for recurrent LOS vs. no LOS; OR 1.7 (1.4–2.3) for recurrent LOS vs. one LOS	Adjusted in multiple logistic regression	p = 0.003 (Bonferroni-Holm corrected pB = 0.012)
Horváth-Puhó et al., [16] (2021)	NDIs, including cognitive delays	NDIs, including motor impairments	NDIs, including sensory impairments	Denmark: RR 1.77 (95% CI 1.44–2.18) for NDIs at 10 years; Netherlands: RR 2.28 (95% CI 1.64–3.17) for NDIs at 10 years; For meningitis mortality: Denmark aHR 4.08 (1.78–9.35), Netherlands aHR 6.73 (3.76–12.06)	Sex, year, and month of birth, gestational age	p<0.0001 for increased outpatient visits and hospital admissions in exposed children
Horváth-Puhó et al., [17] (2022)	NDI reported as a combined outcome; no specific cognitive domains detailed	NR	NR	Preterm children with iGBS had NDI risk: Denmark 8.8%, Netherlands 9.0%; Dutch preterm iGBS mortality rate by 3 months: 671/1000 (95% CI, 412–929/1000) person-years	Analyses adjusted for birth year/month, sex, and gestational age	NR
Golin et al., [18] (2022)	NR	Tone abnormalities, altered tone patterns, abnormal reflexes, abnormal movements	NR	NR	NR	Tone (p < .001), Tone patterns (p = .026), Reflexes (p = .002), Movements (p < .001), Abnormal signs (p < .001), Behavior (p < .001), Total HNNE abnormal in 86% vs. 26% (p < .001)
Brumbaugh et al., [19] (2022)	NDI, including cognitive outcomes	NDI, including motor outcomes	NDI, including sensory outcomes	Adjusted OR for death or NDI in LOM: 1.53 (95% CI, 1.04-2.25); Adjusted OR for death or NDI in LOS without LOM: 1.41 (95% CI, 1.29-1.54)	Adjusted for relevant covariates	LOM: p < .05 los without= p=lom:=
Shim et al., [20] (2021)	Cognitive delay at 18–24 months	Motor delay at 18–24 months	NR	Cognitive delay: OR 1.48 (95% CI 1.02–2.16); Motor delay: No significant association reported	Parental education status, clinical variables (including IVH ≥ grade 3, PVL, IUGR, duration of mechanical ventilation, male sex, NEC ≥ grade 2)	Cognitive delay: p < 0.05 (significant); Motor delay: not significant
Ortgies et al., [21] (2021)	MDI median scores: EOS group 96 (IQR: 86–106) vs. control 94 (84–106)	PDI median scores: EOS group 96 (86–106) vs. control 99.5 (92–103)	NR	NDI in EOS group: 24% vs. control: 8%; OR 3.3	Chorioamnionitis, poor CRIB scores (identified as individual risk factors for MDI or PDI < 70)	MDI p=0.77; PDI p=0.03; NDI OR p=0.03
Mukhopadhyay et al., [22] (2021)	NDI assessed at 18–26 months corrected age	NR	NR	LOS vs. LOCNC for death/NDI: 1.14 (1.05–1.25); LOS vs. LOCNC for death before follow-up: 1.71 (1.44–2.03); LOS vs. LOCNC for NDI among survivors: 0.99 (0.86–1.13); LOCNC vs. unaffected for NDI: 1.17 (1.04–1.31)	Adjusted for covariates occurring ≤7 days of age (specific covariates not detailed in abstract)	LOS vs. LOCNC death/NDI p<0.05; LOS vs. LOCNC death before follow-up p<0.05; LOS vs. LOCNC NDI among survivors not significant; LOCNC vs. unaffected for NDI p<0.05
Nakwa et al., [23] (2020)	24.4% abnormal neurodevelopment (including cognitive) in sepsis cases at 1 year	Included within the overall neurodevelopmental impairment	NR	aOR 3.51 (95% CI: 1.23–10.04)	Gender	0.019

Risk of Bias Findings

The risk of bias assessment using the NOS revealed that most studies (10/14) had a low risk of bias, scoring between 7 and 9 stars, indicating robust methodology in selection, comparability, and outcome assessment. These included Farheen et al. [10], Zhou et al. [11], Paul et al. [13], Humberg et al. [15], Horváth-Puhó et al. [16,17], Brumbaugh et al. [19], Mukhopadhyay et al. [22], and Shim et al. [20], which demonstrated representative cohorts, adequate control for confounders (e.g., gestational age), and reliable outcome measures with minimal attrition. Four studies exhibited a moderate risk (5-6 stars) due to limitations such as incomplete adjustment for confounders [14,18,23] or shorter follow-up [21], primarily losing stars in comparability (C2) or outcome domains (O3). No studies were deemed high-risk, though retrospective designs [12] and cross-sectional analyses [18] warranted cautious interpretation due to potential misclassification or a lack of longitudinal data (Table 3).

**Table 3:** Risk of Bias Using the Newcastle-Ottawa Scale (NOS) Tool Interpretations: Low risk (7-9★), Moderate (5-6★), and High (1-4★) Domain Key: *Selection*: S1: Representativeness of exposed cohort; S2: Selection of non-exposed cohort; S3: Ascertainment of exposure; S4: Demonstration that outcome was not present at baseline ​​​*Comparability*: C1: Control for gestational age/birth weight; C2: Control for additional confounders (e.g., sepsis severity, socioeconomic status) ​​​​​*Outcome/Exposure*: O1: Blind assessment of outcomes; O2: Adequate follow-up duration (>2 years); O3: Completeness of follow-up (>80% retention)

First Author (Year)	Selection				Comparability		Outcome/Exposure			Total (Max 9)	Risk of Bias
	S1	S2	S3	S4	C1	C2	O1	O2	O3		
Farheen et al., [10] (2024)	★	★	★	★	★	★	★	★	★	9	Low
Zhou et al., [11] (2024)	★	★	★	★	★	★	★	★	★	9	Low
Kurul et al., [12] (2023)	★	★	★	★	★	–	★	★	★	8	Low
Paul et al., [13] (2022)	★	★	★	★	★	★	★	★	★	9	Low
Kartam et al., [14] (2022)	★	★	★	–	★	–	★	★	–	6	Moderate
Humberg et al., [15] (2022)	★	★	★	★	★	★	★	★	★	9	Low
Horváth-Puhó et al., [16] (2021)	★	★	★	★	★	★	★	★	★	9	Low
Horváth-Puhó et al., [17] (2022)	★	★	★	★	★	★	★	★	★	9	Low
Golin et al., [18] (2022)	★	★	★	–	★	–	★	★	–	6	Moderate
Brumbaugh et al., [19] (2022)	★	★	★	★	★	★	★	★	★	9	Low
Shim et al., [20] (2021)	★	★	★	★	★	–	★	★	★	8	Low
Ortgies et al., [21] (2021)	★	★	★	–	★	–	★	★	★	7	Low
Mukhopadhyay et al., [22] (2021)	★	★	★	★	★	★	★	★	★	9	Low
Nakwa et al., [23] (2020)	★	★	★	–	★	–	★	★	–	6	Moderate

Discussion

The findings of this review underscore the significant and multifaceted impact of neonatal sepsis on long-term neurodevelopment, particularly in cognitive, motor, and sensory domains. Across the 14 included studies, neonatal sepsis was consistently associated with adverse neurodevelopmental outcomes, with preterm infants and those with severe sepsis or comorbid conditions facing the highest risks. The results align with and expand upon existing literature, highlighting the need for early intervention and long-term follow-up for sepsis survivors.

Cognitive impairments were a prominent finding, with studies such as Farheen et al. [10] and Zhou et al. [11] reporting delays in cognitive milestones and higher rates of sNDI among sepsis survivors. The use of standardized tools, such as the Bayley Scales of Infant Development, revealed that cognitive scores often fell below clinical thresholds (<85), particularly in cases involving meningitis or high sepsis severity scores (e.g., nSOFA ≥4; Kurul et al., [12]). These findings are consistent with prior research demonstrating that neonatal sepsis disrupts critical periods of brain development, leading to long-term deficits in executive function and learning [24]. The elevated risk of cognitive delays in preterm infants with iGBS infections [17] further emphasizes the vulnerability of this population, likely due to compounded effects of prematurity and systemic inflammation on the immature brain [25].

Motor deficits were equally prevalent, with studies such as Kartam et al. [14] and Humberg et al. [15] linking sepsis to cerebellar hemorrhage, reduced motor scores, and cerebral palsy. The association between recurrent LOS and motor deficits in ELBWIs [15] suggests a dose-dependent effect of sepsis severity on motor outcomes, corroborated by animal models showing inflammation-induced damage to motor tracts [26]. Notably, Ortgies et al. [21] reported that EOS impaired psychomotor development, with lower PDI scores independent of cognitive deficits. This dissociation between motor and cognitive outcomes may reflect distinct neuropathological mechanisms, such as white matter injury in motor pathways versus cortical or hippocampal damage in cognitive domains [24].

Sensory outcomes, though less extensively studied, showed mixed results. While Farheen et al. [10] found no significant hearing deficits, Paul et al. [13] included hearing and vision impairments within broader NDI metrics, suggesting sensory deficits may be overshadowed by more severe cognitive or motor impairments. This aligns with literature indicating that sensory impairments in sepsis survivors often co-occur with other NDIs but may be underreported due to assessment limitations [27]. The lack of isolated sensory data in many studies highlights a critical gap, as sensory deficits can exacerbate cognitive and motor challenges by limiting environmental engagement [28].

The role of sepsis severity and comorbidities emerged as a key theme. Kurul et al. [12] demonstrated that inflammatory biomarkers (e.g., CRP) and clinical scores (nSOFA) predicted worse outcomes, supporting the hypothesis that systemic inflammation drives neurotoxicity via cytokine-mediated damage [26]. Similarly, Brumbaugh et al. [19] found that LOM carried higher risks than sepsis alone, likely due to direct central nervous system invasion. These findings mirror meta-analyses linking neonatal infection severity to neurodevelopmental trajectories [29], though our review adds granularity by identifying specific risk factors (e.g., IVH, PVL, Shim et al., [20]) that exacerbate outcomes.

Geographical and methodological diversity across studies enriched the review's applicability. For instance, Farheen et al. [10] and Paul et al. [13] highlighted outcomes in low-resource settings, where limited access to early interventions may amplify deficits. In contrast, studies from high-income countries [16,22,23] have leveraged national registries for long-term follow-up, underscoring the value of standardized surveillance. However, disparities in sepsis definitions and outcome tools (e.g., TQS vs. Bayley Scales) complicate cross-study comparisons, a challenge noted in prior reviews [30].

The risk of bias assessment bolstered confidence in these findings, with 10/14 studies rated as low risk. However, moderate-risk studies [18,23] often lacked confounder adjustment or longitudinal follow-up, echoing critiques of sepsis neurodevelopment literature [31]. Retrospective designs [12] risk misclassification, while cross-sectional studies [18] preclude causal inferences. These limitations underscore the need for prospective, harmonized studies to clarify sepsis-specific effects versus confounding by prematurity or socioeconomic factors [32].

Limitations

This review has several limitations. First, heterogeneity in sepsis definitions (e.g., pSBI vs. culture-confirmed) and outcome measures (e.g., TQS vs. Bayley Scales) precluded meta-analysis, limiting quantitative synthesis. Second, most studies focused on preterm or VLBW infants, potentially underestimating sepsis effects in term neonates. Third, sensory outcomes were underreported, and few studies addressed socioeconomic moderators, despite their known impact on neurodevelopment [33]. Finally, publication bias may favor studies reporting significant associations, though our inclusive search mitigated this risk.

## Conclusions

Neonatal sepsis poses a significant threat to long-term neurodevelopment, with cognitive and motor impairments being the most consistent sequelae. The review highlights the roles of sepsis severity, prematurity, and comorbidities in exacerbating outcomes, while underscoring gaps in sensory and low-resource setting data. These findings advocate for standardized sepsis definitions, routine neurodevelopmental surveillance, and targeted interventions for high-risk infants. Future research should prioritize longitudinal designs and mechanistic studies to disentangle sepsis-specific effects from confounding factors, ultimately guiding precision medicine approaches for this vulnerable population.

## Appendices

**Table 4:** Search Strategy for PubMed, Web of Science, Scopus, and Embase

Database	Search Strategy
PubMed	(“Sepsis”[Mesh] OR sepsis[tiab] OR “neonatal sepsis”[tiab] OR “infant sepsis”[tiab] OR “newborn sepsis”[tiab] OR “neonatal infection”[tiab]) AND (“Infant, Newborn”[Mesh] OR neonat[tiab] OR newborn[tiab] OR “preterm”[tiab] OR “low birth weight”[tiab]) AND (“Neurodevelopmental Disorders”[Mesh] OR neurodevelopment[tiab] OR “cognitive outcome”[tiab] OR “cognitive impairment*”[tiab] OR “sensory outcome”[tiab] OR “hearing loss”[tiab] OR “vision disorders”[tiab]) AND (“Follow-Up Studies”[Mesh] OR “long-term”[tiab] OR “long term”[tiab] OR “outcome”[tiab]) Filters: Publication date from 2020/01/01 to 2025/07/15; Humans; English.
Web of Science	TS=( (sepsis OR "neonatal sepsis" OR "infant sepsis" OR "newborn sepsis" OR "neonatal infection") AND (neonat OR newborn OR "preterm" OR "low birth weight") AND (neurodevelopment OR "cognitive outcome" OR "cognitive impairment" OR "sensory outcome" OR "hearing loss" OR "vision disorders") AND ("long-term" OR "long term" OR outcome) ) Refined by: Document Types = (Article); Languages = (English); Timespan = 2020-2025.
Scopus	TITLE-ABS-KEY ( (sepsis OR "neonatal sepsis" OR "infant sepsis" OR "newborn sepsis" OR "neonatal infection") AND (neonat OR newborn OR preterm OR "low birth weight") AND (neurodevelopment OR "cognitive outcome" OR "cognitive impairment" OR "sensory outcome" OR "hearing loss" OR "vision disorders") AND ("long-term" OR "long term" OR outcome) ) AND (LIMIT-TO (PUBYEAR, 2025) OR LIMIT-TO (PUBYEAR, 2024) OR LIMIT-TO (PUBYEAR, 2023) OR LIMIT-TO (PUBYEAR, 2022) OR LIMIT-TO (PUBYEAR, 2021) OR LIMIT-TO (PUBYEAR, 2020)) AND (LIMIT-TO (LANGUAGE, "English")).
Embase	('neonatal sepsis'/exp OR 'infant sepsis':ab,ti OR 'newborn sepsis':ab,ti OR 'neonatal infection':ab,ti) AND ('neonate'/exp OR neonat:ab,ti OR newborn:ab,ti OR preterm:ab,ti OR 'low birth weight':ab,ti) AND ('neurodevelopmental disorder'/exp OR neurodevelopment:ab,ti OR 'cognitive outcome':ab,ti OR 'cognitive impairment':ab,ti OR 'sensory outcome':ab,ti OR 'hearing loss'/exp OR 'vision disorder'/exp) AND ('long term follow up'/exp OR 'long-term':ab,ti OR 'long term':ab,ti OR outcome:ab,ti) AND (2020-2025)/py AND [english]/lim AND [humans]/lim
